# Retraction: Impact of COVID-19 pandemic on consumer behavioural intention to purchase green products

**DOI:** 10.1371/journal.pone.0341706

**Published:** 2026-01-27

**Authors:** The PLOS One Editors

The *PLOS One* Editors retract this article [1] because it was identified as one of a series of submissions for which we have concerns about potential manipulation of the publication process and authorship. These concerns call into question the validity and provenance of the reported results. We regret that the issues were not identified prior to the article’s publication.

Additionally, contrary to the article’s Data Availability statement, the data underlying the results were not included with the published article. The authors provided the data upon request during editorial follow-up. During editorial review of these data, it was noted that one of the five questionnaire items was removed from the results of the “Fear of COVID-19 Pandemic” questionnaire. The corresponding author stated that one question (‘I feel anxious when thinking about the COVID-19 pandemic’) was dropped because its factor loading was low (0.70), to ensure construct reliability and validity, and they provided citations to justify this exclusion. The Editors consider this concern resolved by the authors’ explanation.

The data provided also indicated that 508 samples were removed from the first raw dataset. The authors responded that these were removed to ensure the validity and reliability of the study, but did not clarify further what parameters were used to determine why and how these samples were removed. The *PLOS One* Editors do not consider this concern resolved.

MAB did not agree with the retraction. MKR did not respond to the final editorial decision. PH, MMH, and SA either did not respond directly or could not be reached.

## References

[1] Hu P, Bhuiyan MA, Rahman MK, Hossain MM, Akter S. RETRACTED: Impact of COVID-19 pandemic on consumer behavioural intention to purchase green products. PLoS One. 2022;17(10):e0275541. doi: 10.1371/journal.pone.0275541 36260619 PMC9581351

